# The GLP-1 Analog Liraglutide Reduces Fever Through Sex-Dependent Neuroinflammatory Modulation

**DOI:** 10.3390/ph18111738

**Published:** 2025-11-15

**Authors:** Gabriela L. Soares de Sousa, Ester K. Martins da Cruz, Sara C. Rojas de Aguiar, Ana P. Lima do Nascimento, Bruna R. Bezerra Gomes, Anna B. Rodrigues Londe, Luana J. Faria Gonçalves, Carine Royer, Regina Azevedo Costa, Aleksander Roberto Zampronio, Paulo Eduardo Narcizo de Souza, Fabiane H. Veiga-Souza

**Affiliations:** 1 Laboratory of Experimental Biomodels, Faculty of Health Sciences and Technology, University of Brasília, Brasília 72220-275, DF, Brazil; gabrielalunaa@hotmail.com (G.L.S.d.S.); esterkarolline2014@gmail.com (E.K.M.d.C.); crhistina_sara@hotmail.com (S.C.R.d.A.); anagomesfarmaceutica@gmail.com (A.P.L.d.N.); beatrizrodrigueslonde@hotmail.com (A.B.R.L.); luanajuliafg@gmail.com (L.J.F.G.); 2 Laboratory of Biochemistry and Protein Chemistry, Institute of Biology, University of Brasília, Brasília 70910-900, DF, Brazil; brunarbgomes@gmail.com; 3 Laboratory of Molecular Pharmacology, Faculty of Health Sciences, University of Brasília, Brasília 70910-900, DF, Brazil; cariroyer@unb.br; 4 Department of Pharmacology, Biological Sciences Section, Federal University of Paraná, Curitiba 81531-980, PR, Brazil; reginaazcosta@hotmail.com (R.A.C.); aleksander@ufpr.br (A.R.Z.); 5 Laboratory of Electron Paramagnetic Resonance, Institute of Physics, University of Brasília, Brasília 70910-900, DF, Brazil; psouza@unb.br

**Keywords:** prostaglandin E2, interleukin-6, serotonin, hypothalamus, lipopolysaccharide, antipyretic

## Abstract

**Background/Objectives**: Thermoregulation is essential for survival, with the hypothalamic preoptic area integrating peripheral signals to maintain core body temperature. While fever enhances immune responses, excessive hyperthermia causes cellular damage. Previous work has shown that central glucagon-like peptide-1 (GLP-1) receptor antagonism intensifies lipopolysaccharide (LPS)-induced fever, suggesting a role for GLP-1 signaling in temperature regulation. However, the direct effects of GLP-1 receptor agonists on fever remained unexplored. This study investigated the effects of liraglutide (LIRA), a GLP-1 analog used to treat diabetes and obesity, on temperature regulation and fever in rats, with a focus on sex-dependent mechanisms. **Methods**: Male and female Wistar rats received lipopolysaccharide (LPS, i.p.) to induce fever, followed by LIRA treatment (0.3 mg/kg, i.p.) one hour later. Body temperature was monitored for up to six hours post-LPS injection. **Results**: LIRA reduced body temperature in both euthermic and febrile rats of both sexes. LPS increased PGE_2_ concentration in both sexes, with males showing a twofold increase compared to females. LIRA treatment reduced PGE_2_ levels in LPS-challenged males (62%, *p* < 0.01) but not in female rats. LPS elevated interleukin (IL)-6 levels in both sexes, while LIRA treatment decreased IL-6 only in females (45%, *p* < 0.05). In males, LPS reduced hypothalamic serotonin (5-HT) levels, and LIRA further decreased 5-HT in saline-treated animals. In females, LIRA increased 5-HT levels (84%, *p* < 0.01) in LPS-challenged animals. Additionally, LIRA exhibited sex-specific effects on hypothalamic JNK phosphorylation, increasing activation in LPS-treated males and reducing it in LPS-treated females. **Conclusions**: LIRA demonstrates antipyretic properties through distinct, sex-specific mechanisms. In males, temperature reduction correlates with decreased hypothalamic PGE_2_, whereas in females, antipyretic effects are associated with reduced IL-6, decreased JNK phosphorylation, and increased 5-HT. These findings reveal sexually dimorphic GLP-1R-mediated thermoregulatory pathways during inflammation. However, the causal relationships between these molecular changes and temperature regulation require further investigation, particularly regarding whether observed biochemical alterations represent primary mechanisms or secondary consequences of temperature modulation. Future studies should investigate the functional significance of the apparent contradiction in serotonergic responses between sexes.

## 1. Introduction

Fever represents a centrally regulated elevation in body temperature that constitutes a fundamental component of the acute-phase response to infectious and inflammatory stimuli [1]. Thermoregulation is critical for organism survival, with the preoptic area of the anterior hypothalamus integrating peripheral signals to maintain core body temperature through activation of heat conservation and dissipation mechanisms [2]. While fever enhances immune responses by promoting leukocyte migration, phagocytosis, and antibody production, excessive hyperthermia imposes substantial metabolic demands, increases oxygen consumption, and can result in protein denaturation and cellular damage [3,4].

The hypothalamic control of fever involves complex neuroinflammatory cascades. Lipopolysaccharide (LPS) administration triggers the release of pro-inflammatory cytokines, including IL-6, which stimulates cyclooxygenase-2 (COX-2) expression in cerebral endothelial cells, leading to prostaglandin (PG) E_2_ synthesis. PGE_2_ subsequently activates EP3 receptors on thermosensitive neurons within the preoptic area, initiating the febrile response. Anti-inflammatory mediators, particularly IL-10, provide regulatory control by inhibiting pro-inflammatory signaling pathways and modulating PG synthesis [2,5].

Sex differences in thermoregulation have been consistently observed in both animals and humans [6,7]. Body temperature patterns fluctuate throughout the ovarian cycle in females, indicating a regulatory role of sex hormones in maintaining thermal homeostasis [8,9]. Evidence shows that randomly cycling female rats exhibit diminished febrile responses compared to males, which stems from the modulatory effects of female gonadal hormones on thermoregulatory and inflammatory pathways [10]. Furthermore, vasopressin antagonism has been found to increase hypothalamic PGE_2_ levels in febrile male rats but not in females, highlighting sex-specific mechanisms involved in fever regulation [11].

Liraglutide (LIRA), a glucagon-like peptide-1 receptor (GLP-1R) agonist, is currently employed in the treatment of type 2 diabetes mellitus and obesity. The growing use of GLP-1 analogs underscores the need to comprehend their effects beyond mere metabolic regulation, particularly as many patients are now exposed to these medications daily. Recent studies have revealed that LIRA possesses anti-inflammatory and neuroprotective properties in various clinical conditions, including osteoarthritis and hepatic ischemia–reperfusion injury [12,13]. The relationship between GLP-1R signaling and the regulation of thermoregulation and fever remains largely uncharted. However, prior research has indicated that blocking central GLP-1R can intensify fever induced by LPS, suggesting a potential involvement of GLP-1 in temperature regulation [14]. Given the prevalent use of GLP-1 analogs and their possible effects on central nervous system function, understanding their influence on body temperature regulation and fever response holds significant clinical importance.

Despite these observations suggesting GLP-1 involvement in temperature regulation, the direct antipyretic effects of LIRA during systemic inflammation remain uncharacterized, and the sex-specific mechanisms underlying potential thermoregulatory responses have not been investigated. This study addressed these gaps by examining LIRA’s effects on body temperature in male and female rats during LPS-induced fever, its influence on hypothalamic and systemic inflammatory and oxidative markers, and the role of neurotransmitters such as serotonin (5-HT) and dopamine (DA). We also investigated sex-specific effects of LIRA on hypothalamic JNK phosphorylation to clarify the molecular mechanisms underlying GLP-1 receptor-mediated modulation of fever.

## 2. Results

### 2.1. LIRA Reduces Body Temperature in Both Euthermic and Febrile Rats

**Figure 1:**
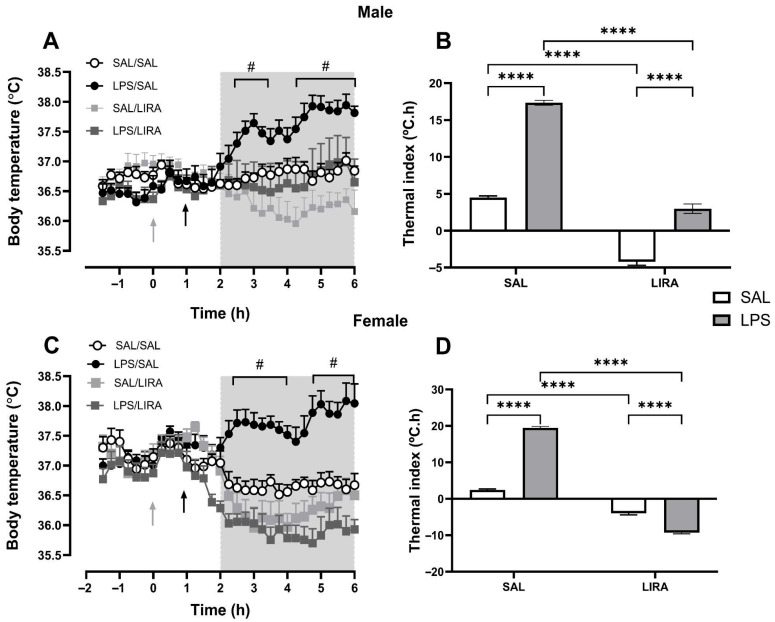
LIRA reduces the febrile response induced by LPS in male and female rats. Rats were treated with LIRA via intraperitoneal injection (i.p.) at a dose of 0.3 mg/kg, or with saline (SAL) at a volume of 0.5 mL, administered 1 h after an i.p. injection of LPS (50 μg/kg) or sterile saline. (**A**,**C**) The data are expressed as mean ± SEM of body temperature (°C) in 6–7 animals. The gray and black arrows represent the time of LPS and LIRA injections, respectively. (**B**,**D**) Thermal index calculated from the area under the curve, from 2 to 6 h (as indicated in panels **A**,**C**). **** *p* < 0.0001, # *p* < 0.05 LPS/SAL compared with SAL/SAL. Data were analyzed by (**A**,**C**) three-way ANOVA matching by factor time and (**B**,**D**) two-way ANOVA, both followed by Tukey’s multiple comparison test.

To investigate the effect of LIRA on fever, we first injected LPS intraperitoneally, and after 1 h, we administered the treatment with LIRA (0.3 mg/kg) by the same route to animals of both sexes. As expected, LPS administration evoked a significant increase in body temperature compared with the saline-treated group, which maintained a relatively stable temperature, with minor fluctuations likely due to animal handling. LIRA administration completely abolished LPS-induced fever (Figure 1A,C). Interestingly, the temperature of euthermic animals (SAL/LIRA group) was also reduced by LIRA treatment in both male and female rats (Figure 1A,C).

To quantify these effects, thermal index values were calculated from the area under the curve during the gray-shaded period, providing clearer quantification of the time-course results shown in Figure 1A,C. It was observed sex-specific differences in LIRA’s effects. In males (Figure 1B), the SAL/LIRA group demonstrated a greater reduction in thermal index compared to the LPS/LIRA group, indicating that LIRA’s hypothermic effect was attenuated in the presence of LPS-induced inflammation. Conversely, in females (Figure 1D), the LPS/LIRA group exhibited a more pronounced reduction in thermal index than the SAL/LIRA group, suggesting that LIRA’s temperature-lowering effects were enhanced during the inflammatory state. LIRA reduced thermal index by approximately 83% in LPS-challenged males (LPS/LIRA vs. LPS/SAL). In females, LIRA not only abolished fever but induced hypothermia, lowering the thermal index by 148% and reducing body temperature below baseline.

### 2.2. Effect of Post-Treatment with LIRA on Hypothalamic PGE_2_ Production

**Figure 2:**
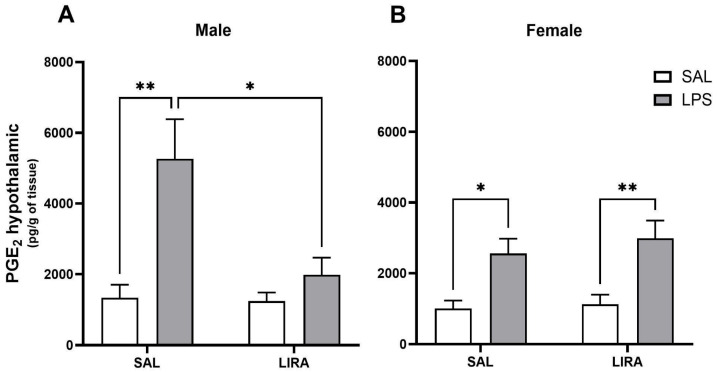
LIRA attenuates LPS-induced elevation of hypothalamic PGE_2_ in male rats. (**A**,**B**) show the concentration of PGE_2_ per gram of hypothalamic tissue collected 4 h after LPS administration in male and female rats, respectively. Data represent mean ± SEM and were analyzed using two-way ANOVA followed by Tukey’s multiple comparison test. * *p* < 0.05; ** *p* < 0.01.

To investigate the mechanism by which LIRA reduces the body temperature of these animals, we measured the hypothalamic concentration of PGE_2_. Intraperitoneal administration of LPS increased PGE_2_ concentration in both male and female rats, with the concentration being twice as high in males as in females (Figure 2A,B). LIRA treatment reduced PGE_2_ concentration in male rats challenged with LPS by 62% (*p* < 0.01) (Figure 2A). In contrast to males, LIRA treatment did not alter PGE_2_ concentration in female rats (Figure 2B), suggesting sex-specific mechanisms underlying LIRA’s antipyretic effect.

### 2.3. Effect of Post-Treatment with LIRA on Circulating IL-6 and IL-10 Levels

**Figure 3:**
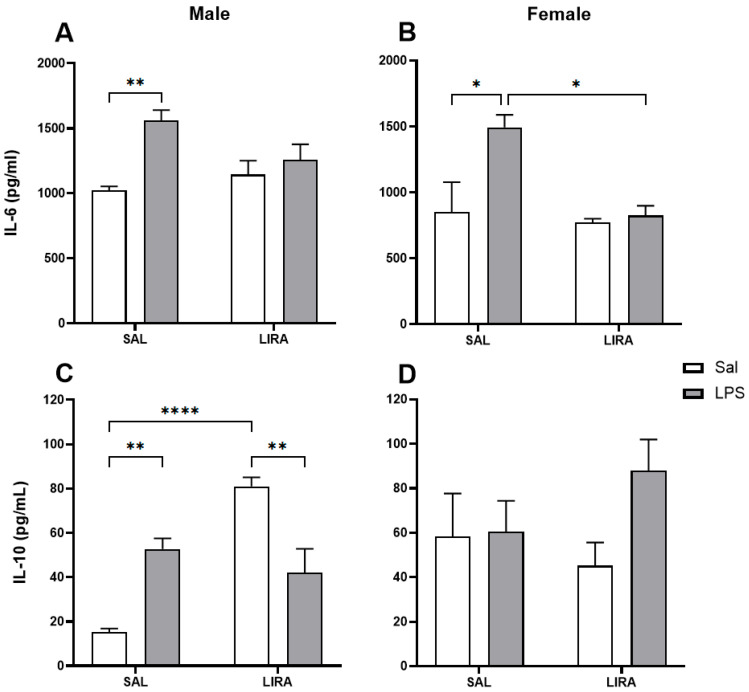
Serum concentrations of IL-6 and IL-10 after LPS administration in rats treated with or without LIRA. Panels (**A**,**B**) show IL-6 concentrations in male and female rats, respectively. Panels (**C**,**D**) show IL-10 concentrations in male and female rats, respectively. Animals were divided into control (SAL) and LIRA-treated groups (0.3 mg/kg, i.p.) and evaluated 4 h after administration of LPS (50 μg/kg, i.p.) or sterile saline (0.9%, 0.5 mL) (*n* = 5 per group). Data are presented as mean ± SEM. Statistically significant differences were indicated by * *p* < 0.05, ** *p* < 0.01, **** *p* < 0.0001, as determined by two-way ANOVA followed by Tukey’s post hoc test.

The proinflammatory cytokine IL-6 was measured in serum to assess the inflammatory response to LPS and the effect of LIRA administration. LPS triggered an increase in circulating IL-6 levels compared to the control group in both male and female rats (Figure 3A,B). LIRA treatment affected only female rats, reducing serum IL-6 levels by 45% (*p* < 0.05). We also measured the concentration of IL-10, an anti-inflammatory cytokine, in these animal sera (Figure 3C,D). LPS caused an increase in the concentration of IL-10 compared to the control group only in male rats. Interestingly, treatment with LIRA promoted an increase in IL-10 levels in saline-treated animals, while it did not change IL-10 levels in LPS-treated animals (Figure 3C). Unlike males, female rats exhibited a markedly different pattern. In female rats, neither LPS administration nor LIRA treatment altered the serum concentration of IL-10 (Figure 3D).

### 2.4. Effect of LIRA on ROS and HbNO Production

**Figure 4:**
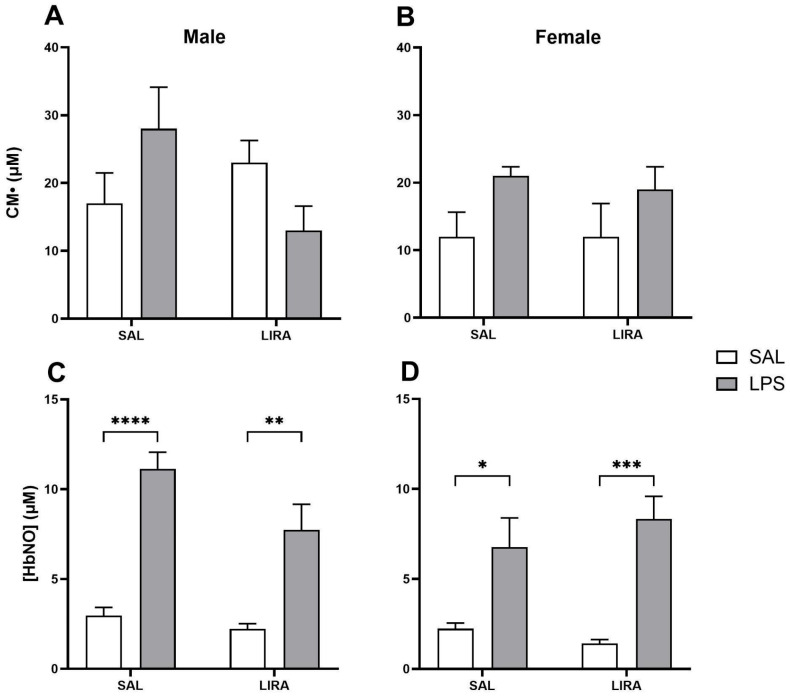
CM• and peripheral HbNO concentrations after LPS administration in rats treated with or without LIRA. Panels (**A**,**B**) show CM• concentrations in male and female rats, respectively. Panels (**C**,**D**) show peripheral HbNO concentrations in males and females, respectively. Animals were divided into control (SAL) and LIRA-treated groups (0.3 mg/kg, i.p.) and evaluated 4 h after administration of LPS (50 μg/kg, i.p.) or sterile saline (0.9%, 0.5 mL) (*n* = 6–7 per group). Data are presented as mean ± SEM. Statistically significant differences were indicated by * *p* < 0.05, ** *p* < 0.01, *** *p* < 0.001, **** *p* < 0.0001, as determined by two-way ANOVA followed by Tukey’s post hoc test.

We investigated the effects of LIRA on blood markers of oxidative stress, i.e., reactive oxygen species (ROS), and nitrosative stress, such as nitrosylated hemoglobin (HbNO). No significant increases in ROS were observed after this dose of LPS in either male or female rats, and LIRA treatment did not alter ROS formation in either sex (Figure 4A,B).

HbNO formation increased significantly after LPS challenge in both male and female rats. However, LIRA treatment did not alter HbNO levels in animals of either sex (Figure 4C,D).

### 2.5. Changes in 5-HT and DA Levels in the Hypothalamus by LIRA

**Figure 5:**
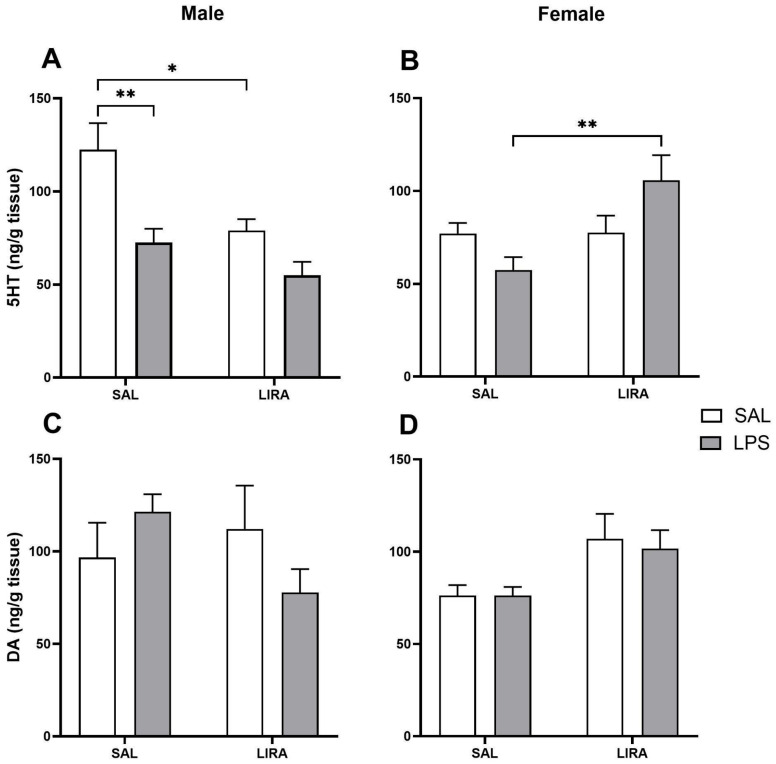
Levels of 5-HT and DA in the hypothalamus after LPS administration in rats treated with or without LIRA. Panels (**A**,**B**) show levels of 5-HT in male and female rats, respectively. Panels (**C**,**D**) show levels of DA in males and females, respectively. Animals were divided into control (SAL) and LIRA-treated groups (0.3 mg/kg, i.p.) and evaluated 4 h after administration of LPS (50 μg/kg, i.p.) or sterile saline (0.9%, 0.5 mL) (n = 6–7 per group). Data are presented as mean ± SEM. Statistically significant differences were indicated by * *p* < 0.05, ** *p* < 0.01, as determined by two-way ANOVA followed by Tukey’s post hoc test.

To investigate whether the body temperature reduction induced by LIRA occurs through a mechanism involving 5-HT or DA, we quantified the levels of these neurotransmitters in the hypothalamus of male and female rats (Figure 5). In male rats, a reduction in 5-HT levels was observed after administration of LPS (Figure 5A). Regarding the treatment, LIRA caused a significant decrease in 5-HT levels by 35% (*p* < 0.05) in saline-treated animals, but did not change the reduction promoted by LPS (Figure 5A). In female rats, LPS administration did not induce changes in 5-HT levels compared to the control group. Interestingly, treatment with LIRA increased 5-HT levels by 84% (*p* < 0.01) in LPS-challenged animals (Figure 5B).

Regarding DA levels, no significant changes were observed after LPS administration or LIRA treatment, either in male or female rats (Figure 5C,D).

### 2.6. Sex-Specific Modulation of Hypothalamic JNK Signaling by LIRA

**Figure 6:**
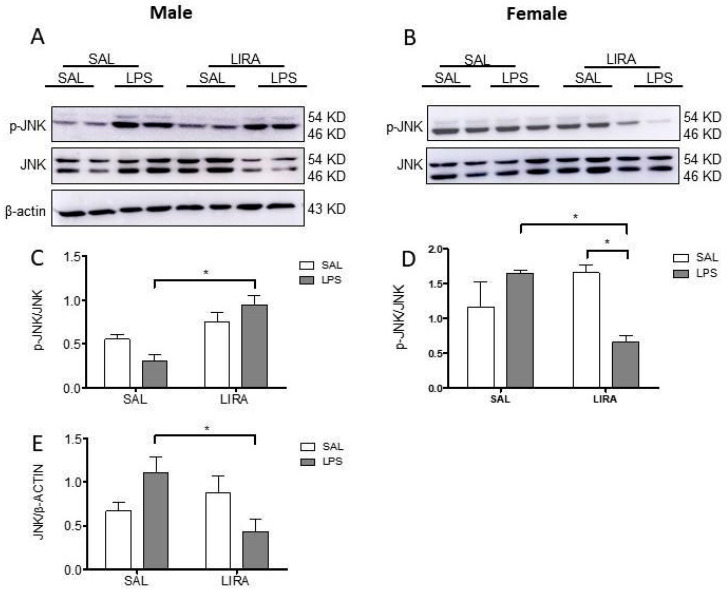
Sex-specific effects of LIRA on hypothalamic JNK phosphorylation in male and female rats following LPS-induced systemic inflammation. Representative Western blot images showing phosphorylated JNK (p-JNK), total JNK, and β-actin protein expression in hypothalamic tissue from male (**A**) and female (**B**) rats. Animals were treated with SAL or LIRA followed by SAL or LPS (50 μg/kg, i.p.). Quantitative analysis shows the ratio of p-JNK to total JNK in males (**C**) and females (**D**), as well as total JNK normalized to β-actin in males (**E**). Data are presented as mean ± SEM (*n* = 6–8 per group). * *p* < 0.05 as determined by two-way ANOVA followed by Tukey’s post hoc test. Molecular weights are indicated on the right side of the blots.

We then examined the molecular pathways involved in the antipyretic effect of LIRA. Given the central role of JNK signaling in inflammatory responses, we investigated the impact of LIRA on hypothalamic JNK phosphorylation in both male and female rats after LPS administration. Western blot analysis revealed sex-dependent differences in JNK activation patterns. In male rats, LPS administration alone did not significantly alter JNK phosphorylation compared to saline controls. However, LIRA treatment in LPS-challenged males resulted in a significant increase in phosphorylated JNK levels (*p* < 0.05), suggesting enhanced kinase activation (Figure 6A,C). Total JNK protein expression showed a corresponding decrease in the LPS/LIRA group compared to LPS/SAL (*p* < 0.05), indicating altered protein turnover or synthesis (Figure 6E).

In contrast, female rats exhibited a markedly different response pattern. Although LPS administration alone did not significantly increase JNK phosphorylation compared to saline controls, LIRA treatment in LPS-challenged females dramatically reduced phosphorylated JNK levels compared to the LPS/SAL group (*p* < 0.05) (Figure 6B,D). Total JNK protein levels remained stable across all female treatment groups, indicating that the observed changes reflect altered kinase activation rather than modifications in protein expression (Figure 6B).

## 3. Discussion

In this study, we investigated the effects of LIRA on central thermoregulatory mechanisms and fever responses induced by systemic inflammation triggered by LPS. We demonstrated that LIRA reduces body temperature in both euthermic and febrile animals of both sexes; however, the mechanisms involved in LIRA’s antipyretic effects are sex-specific.

Fever is generated by the action of several autonomic responses, including peripheral vasoconstriction, decreased sweating, reduced heat loss, shivering, and possibly also non-shivering thermogenesis [5]. In this study, we used a dose of 50 µg/kg intraperitoneally to induce fever in male and female animals. This dose caused a fever of similar magnitude in animals of both sexes, which differs from some studies that demonstrate that cycling females exhibit a fever of lesser magnitude than ovariectomized females or males [10,15]. It is known that estrogen reduces the LPS-induced febrile response [10] and that the production of this hormone varies according to the estrous cycle in female rats [16]. Thus, this discrepancy may be explained by the lack of estrous cycle monitoring in females, which may have masked sex-dependent differences in fever magnitude.

Our findings demonstrate that LIRA reduced body temperature in both euthermic and febrile conditions across male and female animals. These results align with previous research by [14], who demonstrated that central antagonism of GLP-1R increased LPS-induced fever in their experimental model. Additionally, a prior study showed that LIRA attenuates peripheral inflammatory responses, specifically reducing carrageenan-induced paw edema and local temperature in rats [17]. Given that LIRA functions as a GLP-1R agonist, the antipyretic effect observed in our study represents the expected pharmacological response, likely mediated through both central thermoregulatory mechanisms and peripheral anti-inflammatory actions, which suggests the role of GLP-1 signaling in controlling both inflammatory responses and thermoregulation.

The hypothermic effect of LIRA in euthermic animals warrants consideration of both central and peripheral mechanisms. While LIRA can cross the blood–brain barrier and activate central GLP-1 receptors in thermoregulatory centers, peripheral vasodilation via GLP-1R activation in endothelial cells may also contribute to this effect [18,19]. Future experiments, using central versus peripheral administration or vascular imaging techniques, could help dissect these relative contributions.

PGs are considered the final mediators of the febrile response [5]. To elucidate the mechanism by which LIRA reduces fever, we quantified the concentration of PGE_2_ in the hypothalamus of these animals. The results showed that LPS induced an increase in PGE_2_ concentrations in both male and female rats, with the concentration in males being twice that observed in females. In agreement with our data, Brito et al. ref. demonstrated that PGE_2_ production in ovariectomized rats is greater than in cycling female rats after LPS challenge [10]. Interestingly, LIRA treatment reduced the concentration of this mediator only in male animals. These results suggest that the mechanism by which LIRA reduces body temperature in febrile male animals involves a reduction in PGE_2_ concentrations in the hypothalamus. In agreement with our data, previous studies have demonstrated that LIRA can reduce PGE_2_ production after LPS stimulation. A study evaluated the anti-inflammatory effects of LIRA on LPS-activated macrophages, showing that LIRA dose-dependently reduces PGE_2_ production [20]. Another group demonstrated that exendin-4, a GLP-1R agonist with a mechanism of action similar to LIRA, inhibits the expression of the inflammatory mediators iNOS, COX-2, PGE_2_, and NO, as well as pro-inflammatory cytokines in an in vitro model of LPS-induced inflammation in macrophages [21]. Indeed, a recent study published in 2022 demonstrated that, in an osteoarthritis model, LIRA treatment significantly reduced PGE_2_ secretion in vitro in chondrocytes and macrophages [12].

Regarding female animals, we observed no significant difference in PGE_2_ production after LIRA treatment, despite a reduction in body temperature. Indeed, a study demonstrated that female rats may use different strategies to control fever compared to male rats. For example, they observed that a vasopressin antagonist elevates PGE_2_ in febrile male rats, but not in females, showing that PGE_2_ levels in fever may differ between the sexes, which is in agreement with our results [11].

It is well known that LPS administration induces the release of IL-6, a key pyrogenic cytokine involved in the regulation of fever. Its critical role is demonstrated by the fact that direct administration of IL-6 into the brain elicits fever [22]. In contrast, neutralization of this cytokine or its genetic absence prevents the development of the febrile response [3,23,24]. In the present study, LPS increased serum IL-6 levels in both sexes; however, LIRA significantly reduced this cytokine only in females. In agreement, a study in male mice showed that another GPL-1 analogue, exendin-4, did not alter LPS-induced plasma IL-6 production [25]. Conversely, GLP-1 analogs reduced IL-6 in other contexts: exendin-4 in LPS-stimulated macrophages [21], LIRA in osteoarthritis [12], and hepatic ischemia–reperfusion in male mice [13]. Notably, these studies lacked sex-stratified analyses. The female-specific IL-6 reduction observed here, contrasting with male-only studies [13,23], provides strong evidence for sex-dependent responses. This aligns with clinical data showing 32% higher liraglutide exposure in women [26], suggesting our 0.3 mg/kg dose was sufficient to modulate IL-6 in females but not males.

IL-10 is an anti-inflammatory cytokine essential for regulating the immune response, acting to suppress the production of pro-inflammatory cytokines. In our study, we identified increased IL-10 production only in male rats after LPS challenge. Interestingly, LIRA treatment exhibited two distinct effects on IL-10 production depending on the inflammatory context. In control animals from the saline group, LIRA increased IL-10 production, whereas in LPS-treated animals, LIRA did not alter IL-10 production. Indeed, one study demonstrated that in rats with acute inflammation treated with LIRA, IL-10 levels increased dramatically in inflamed paw tissue compared to non-inflamed paws [17]. Another study demonstrated that LIRA alleviated hyperalgesia associated with chronic migraine by inhibiting the levels of CGRP, phosphorylated ERK (p-ERK), and c-fos in the trigeminal nucleus caudalis, while simultaneously increasing IL-10 release [27].

In female rats, we observed basal levels of IL-10 at least 3 times higher than those observed in male rats. However, no significant changes in IL-10 levels were observed in females after LPS administration, which may indicate a sexually dependent response to LIRA. In agreement, one study showed that LIRA promoted increased expression of the anti-inflammatory mediators IL-10 and TGFβ1 in the hippocampi of males, but not in females, of food-restricted mothers [28].

Several studies demonstrate that LIRA attenuates oxidative stress in tissues, such as the liver and heart, as well as in human umbilical vein endothelial cells, possibly through its anti-inflammatory effects [29,30,31]. In this investigation, we hypothesized that the reduction in body temperature caused by LIRA could be associated with a decrease in the production of reactive oxygen species (ROS). As a result, we did not observe a significant increase in ROS in the blood of animals with fever. Treatment with LIRA did not change blood ROS levels. These data are consistent with a previous study, which demonstrated a significant increase in ROS formation in the liver, brown adipose tissue, and hypothalamus, but not blood during the febrile response [32].

Nitric oxide (NO) is a crucial radical involved in various physiological and pathological processes. When there is an increase in NO availability in the blood, nitrosated hemoglobin HbNO is formed, and the reaction of hemoglobin with NO is a protective mechanism for the body during oxidative stress [33]. Therefore, we decided to test the hypothesis that treatment with LIRA would also modulate HbNO concentrations, given that previous studies have shown that NO is involved in the febrile response and acts as an antipyretic molecule during LPS-induced fever [34,35,36]. In our study, animals of both sexes with fever showed an increase in HbNO concentrations in the blood 4 h after LPS injection, indicating that NO availability in the blood increases during fever. However, LIRA treatment did not alter HbNO concentration during fever, demonstrating that the temperature reduction caused by LIRA is not associated with changes in HbNO. In contrast, Helmstädter et al. (2021) concluded that LIRA exerts an anti-inflammatory effect on vascular endothelial cells, through decreased expression of iNOS, IL-6, and 3-nitrotyrosine [37].

Recent evidence suggests that LIRA has the potential to reduce depressive symptoms, and this effect may be partially due to the modulation of central neurotransmitter systems [38]. Given the well-established roles of 5-HT and DA in mood regulation and the alterations in these neurotransmitters observed during systemic inflammation, we investigated the effects of LIRA on hypothalamic levels of 5-HT and DA. This investigation is particularly relevant since 5-HT exhibits antipyretic properties, suggesting that LIRA’s fever-reducing effects may be mediated, at least in part, through increased 5-HT concentrations in thermoregulatory centers.

Sexual dimorphism in the serotonergic system is well established in thermoregulation and inflammatory responses. Previous research found that hypothalamic 5-HT decreases in male rats and increases in female rats during LPS-induced systemic inflammation [15]. In our study, LPS reduced hypothalamic 5-HT in males by 40%, consistent with these findings. However, we did not observe a significant increase in 5-HT in LPS-challenged females. Notably, LIRA treatment produced distinct sex-specific effects: in males, LIRA decreased 5-HT by 36% in euthermic animals and did not further affect the LPS-induced reduction; in females, LIRA increased 5-HT by 84% only in LPS-challenged animals. These results suggest that GLP-1R activation interacts with inflammatory signaling in a sex-dependent manner.

These differing serotonergic responses likely result from estrogen-mediated modulation of serotonergic neurotransmission. Estrogen increases tryptophan hydroxylase expression and activity and reduces 5-HT reuptake through serotonin transporter modulation [39,40]. This regulation may explain why LIRA increased hypothalamic 5-HT only in inflamed females, where inflammatory and estrogenic signals combine to amplify serotonergic responses. In males, where both LPS and LIRA decreased 5-HT, the lack of estrogenic modulation suggests that other mechanisms, such as increased monoamine oxidase activity, enhanced 5-HT turnover and release during inflammation, or altered neuronal firing, may predominate.

The increase in 5-HT in LIRA-treated females corresponds with the observed antipyretic effect, as serotonergic activation reduces fever [41]. This supports a mechanistic role for 5-HT in LIRA’s temperature-lowering effects in females. In males, the antipyretic effect appears independent of 5-HT modulation and is likely due to PGE_2_ reduction. Consistent with our results, a previous study reported decreased plasma 5-HT in male mice after LIRA administration [42].

Regarding DA, a previous study demonstrated a decrease in this neurotransmitter during systemic inflammation in both male and female rats [15]. However, in our study, no changes were observed after the administration of LPS, nor after treatment with LIRA.

Stress-activated protein kinase (JNK) plays a crucial role in inflammatory signaling cascades, being activated by pro-inflammatory stimuli, including LPS [19,43]. LIRA exhibited sex-specific effects on hypothalamic JNK phosphorylation, increasing activation in LPS-treated males while reducing it in LPS-treated females. These opposing responses suggest different molecular mechanisms underlying GLP-1 receptor-mediated neuroimmune modulation, likely arising from regional heterogeneity in GLP-1R signaling across hypothalamic nuclei. Both the preoptic area, which regulates thermoregulation, and the arcuate nucleus, which integrates metabolic signals including leptin, insulin, and glucose, express GLP-1R [44]. Sex differences in receptor density, downstream signaling pathways, or interactions with steroid hormone receptors in these anatomically and functionally distinct regions may account for the observed dimorphism.

**Figure 7:**
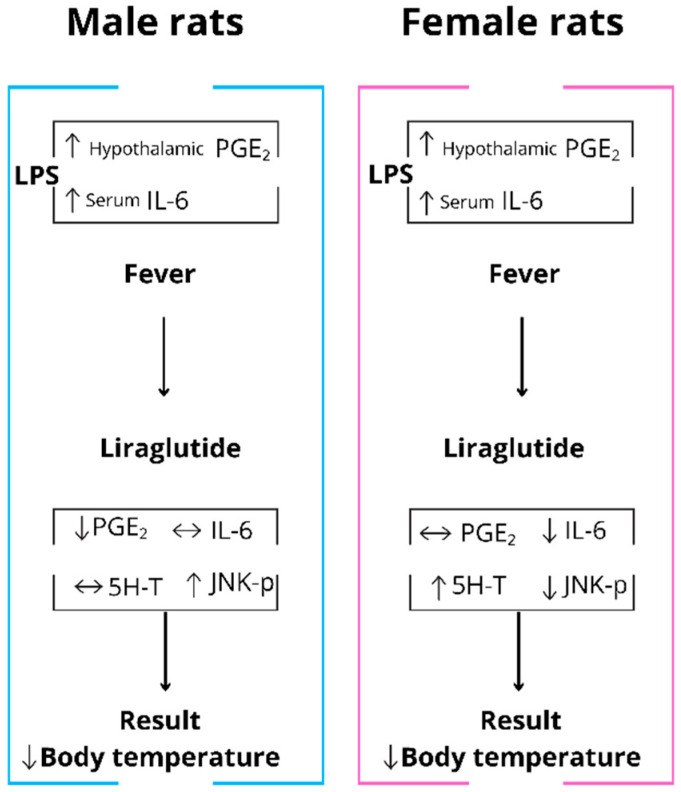
Sex-specific mechanisms underlying LIRA’s antipyretic effects during LPS-induced systemic inflammation. In male rats (left panel), LPS administration increases both hypothalamic PGE_2_ and serum IL-6, inducing fever. Liraglutide treatment reduces body temperature primarily through decreased hypothalamic PGE_2_, while increasing JNK phosphorylation and showing no effect on serotonin (5-HT) or IL-6 levels. In female rats (right panel), LPS similarly elevates hypothalamic PGE_2_ and serum IL-6, producing fever of comparable magnitude. However, liraglutide’s antipyretic effect operates through distinct pathways: reduced serum IL-6, increased hypothalamic 5-HT, and decreased JNK phosphorylation, with no significant change in PGE_2_. Arrows indicate direction of change: ↑ increase, ↓ decrease, ↔ no significant change. 5-HT, serotonin; IL-6, interleukin-6; JNK-p, phosphorylated c-Jun N-terminal kinase; LPS, lipopolysaccharide; PGE_2_, prostaglandin E_2_.

Figure 7 summarizes these sex-specific mechanisms, illustrating how LIRA produces divergent antipyretic pathways in males versus female rats.

While our findings demonstrate significant associations between LIRA treatment and changes in hypothalamic mediators, further experiments are needed to establish causality. Pharmacological blockade using the selective GLP-1R antagonist exendin(9-39) would confirm whether the observed antipyretic and neuroinflammatory effects are mediated by GLP-1R activation. Additionally, immunohistochemical localization of GLP-1R in specific thermoregulatory nuclei would help identify the anatomical basis for these sex-specific responses and clarify the differing signaling patterns between males and females.

Another consideration in interpreting our findings is the single time point (4 h post-LPS) used for molecular analyses. While this captures sustained fever and allows adequate LIRA exposure, it precludes detailed temporal resolution of the cascade linking drug administration to molecular changes and temperature modulation. Future studies employing multiple time points would better establish whether the observed biochemical changes precede, coincide with, or follow temperature reduction, thereby helping to distinguish primary mechanisms from secondary consequences.

These findings have potential clinical implications given the widespread use of GLP-1 analogues in treating type 2 diabetes and obesity, conditions frequently associated with chronic low-grade inflammation. The demonstration that LIRA exerts antipyretic effects through sex-specific mechanisms suggests these medications could influence thermoregulation in clinical populations, warranting systematic investigation. Patients undergoing GLP-1 therapy may experience altered fever responses during infections or inflammatory episodes, and the sex-dependent mechanisms we identified could contribute to documented sex differences in GLP-1 analogue efficacy and tolerability [26]. Prospective clinical studies that monitor body temperature during GLP-1 therapy, with stratification by biological sex, are needed to determine whether these antipyretic mechanisms translate to clinically significant thermoregulatory changes. Clarifying these effects is particularly important for diabetic patients with concurrent systemic inflammation, in whom both metabolic and thermoregulatory homeostasis are compromised.

## 4. Materials and Methods

### 4.1. Animals

This study was conducted at the Laboratory of Protein Chemistry and Biochemistry of the University of Brasilia (UnB) and was approved by the UnB Ethics Committee for the use of Animals (CEUA) (Protocol No. 23106.128494/2022-27). Male and female Wistar rats (8–9 weeks) were obtained from the Evangelical University of Goiás (Anápolis, Goiás, Brazil). The animals were housed four per cage in a ventilated rack (Alesco Indústria e Comércio Ltda, Monte Mor, São Paulo, Brazil) in a temperature-controlled room at 22 ± 1 °C and subjected to a 12 h light–dark cycle (lights on at 7 a.m.), with water and food available ad libitum.

### 4.2. Drugs

LPS from Escherichia coli (0111: B4, Sigma-Aldrich, St. Louis, MO, USA) was diluted in sterile saline and administered i.p. at a dose of 50 µg/kg. LIRA (Saxenda^®^, Novo Nordisk, Bagsværd, Denmark) was diluted in sterile saline and administered i.p. at a dose of 0.3 mg/kg. The doses of LPS and LIRA were selected based on pilot experiments and previous studies [10,17,45]. Ketamine, xylazine, and acepromazine were purchased from Vetnil (Louveira, SP, Brazil). Oxytetracycline (Terramicina^®^, Zoetis, SP, Brazil) and meloxicam (Maxicam^®^, MSD, SP, Brazil) were of commercial grade.

### 4.3. Temperature Datalogger Implantation and Core Temperature Measurements

Animals were anesthetized with a mixture of xylazine at 10 mg/kg, ketamine hydrochloride at 60 mg/kg, and acepromazine at 1 mg/kg, administered intraperitoneally. Incisions were made in the skin and peritoneal muscle to implant temperature dataloggers (Subcue, Calgary, AB, Canada), followed by suturing of the muscle and skin. Post-surgery, animals received an intramuscular injection of oxytetracycline at 10 mg/kg and a subcutaneous injection of meloxicam at 1 mg/kg. Meloxicam administration was repeated for two additional days. Following these procedures, animals were returned to their cages for a seven-day recovery period before pharmacological experiments began.

### 4.4. Experimental Design

Experiments were conducted in the rats’ thermoneutral zone (27 ± 1 °C) (Gordon, 1990) [46], after animals had acclimated to the environment for at least 12 h. Body temperatures were monitored at 15 min intervals throughout the experiments. To investigate LIRA’s effects on fever, rats received an injection of LPS (50 µg/kg, i.p.) to induce fever, followed by post-treatment with LIRA (0.3 mg/kg) 1 h later. Animals had their body temperature monitored for 6 h following LPS administration. For experiments involving the collection of hypothalamic tissue and blood samples for analysis, the same experimental design was followed, with animals being euthanized 4 h after LPS administration. This time point was selected to capture sustained inflammatory responses while allowing 3 h of LIRA exposure, providing adequate time for the drug to exert its pharmacological effects. The animals were then euthanized, the hypothalami were immediately dissected, immersed in liquid nitrogen, and stored at −80 °C until analysis. Blood samples were also collected for the quantification of reactive oxygen species (ROS) and nitrosylated hemoglobin (HbNO), while serum was obtained for the measurement of IL-6 and IL-10 concentrations. All biological samples were stored at −80 °C until they were analyzed.

### 4.5. Quantification of Hypothalamic PGE_2_ by ELISA

Hypothalamic PGE_2_ concentrations were measured according to the protocol described by [47]. Immediately after collection, samples received 40 μL of indomethacin (1 mg/mL, pH 7.4). The hypothalami were homogenized in 1 mL of RPMI containing indomethacin in Tris-HCl, then acidified with HCl to a pH of 3.5–4.0. After ice incubation, samples were centrifuged at 20,000× *g* (4 °C, 10 min). Supernatants were applied to Sep-Pak C18 cartridges, and PGE_2_ was eluted with 2 mL absolute ethanol. Following 18 h speed vacuum drying, pellets were resuspended in EIA buffer (supplied with the kit) and analyzed using Cayman Chemical’s Prostaglandin E_2_ ELISA Kit (detection limit: 7.8 pg/mL, Cayman Chemical, Ann Arbor, MI, USA). 

### 4.6. Measurement of IL-6 and IL-10 Levels in Serum

For IL-6 and IL-10 quantification, blood samples were collected via cardiac puncture during exsanguination in heparinized tubes and centrifuged for 10 min at 4700× *g* at 4 °C. The supernatant was transferred to new tubes and stored at −80 °C until further analysis. IL-6 and IL-10 production were evaluated by ELISA, carried out according to the manufacturer’s instructions (Catalog Number RAB0311 and RAB0246, respectively, Sigma Aldrich, St. Louis, MO, USA). First, standard curves were performed using reagents provided by the kit, and the serum levels of the cytokines were determined according to this curve.

### 4.7. Quantification of ROS and HbNO by EPR Spectroscopy

ROS and HbNO were quantified in blood using electron paramagnetic resonance (EPR) spectroscopy following established methodology [32,33]. For ROS quantification, the spin trap CMH (10 mM) was dissolved in freshly prepared Krebs-HEPES buffer (KHB) containing deferoxamine (25 mM) and sodium diethyldithiocarbamate trihydrate (5 mM) as metal chelators. CMH is oxidised, mainly by the superoxide ion, forming the EPR-detectable nitroxide radical CM^•^ that has a half-life of several hours [32]. Blood samples were mixed with equal volumes of 400 μM CMH solution containing sodium heparin (100 IU/mL) and incubated at 37 °C for 30 min with constant agitation. Calibration standards used 3-carboxy-proxyl (CP^•^) at 0, 5, 10, 50, and 100 μM concentrations. For HbNO quantification, cardiac blood was collected 4 h post-LPS via right ventricular puncture using heparinized syringes. After centrifugation (2000× *g*, 5 min), samples were immediately frozen in liquid nitrogen. Calibration curves were generated using erythrocytes treated with nitrite (1, 10, 100 μM) and sodium dithionite (20 mg) as reducing agent. All samples were placed in 1 mL syringes, frozen in liquid nitrogen, and stored at −80 °C. EPR analysis used an EMX Plus spectrometer (X-band, 9 GHz, Bruker, Karlsruhe, Germany) with parameters: 10 mW microwave power, 5 Gauss modulation amplitude, 100 kHz modulation frequency, 12 s scan time, and 4-scan averaging across 240 Gauss sweep width. All reagents were obtained from Noxygen (Elzach, Germany).

### 4.8. Neurotransmitter Measurement by HPLC

Hypothalamic 5-HT and DA concentrations were measured using HPLC with electrochemical detection. Tissue samples were sonicated in 0.1 M perchloric acid containing 0.02% sodium metabisulfite and 3,4-dihydroxybenzylamine (50 ng/mL) as internal standard. After centrifugation (10,000× *g*, 20 min, 4 °C), 20 μL of supernatant was analyzed. The mobile phase consisted of citric acid monohydrate (20 g), octane-1-sulfonic acid sodium salt (200 mg), EDTA (40 mg), and deionized water (900 mL), filtered through 0.45 μm membranes, with methanol added to 10% *v*/*v*. Chromatographic separation used a Synergi Fusion-RPC-18 column (150 × 4.6 mm, 4 μm) with SecurityGuard pre-column at 25 °C. A Coulochem III electrochemical detector (ESA Inc., Chelmsford, MA, USA) with dual-electrode analytical cell (ESA 5011A) was employed, with oxidation potentials of 100 mV and 450 mV. A guard cell (ESA 5020) was set at 350 mV. Concentrations were calculated from standard curves and expressed as ng/g of tissue based on previous studies [15,48].

### 4.9. Western Blotting for Detection of JNK and Phospho-JNK

To detect total and phosphorylated JNK, hypothalamic protein extraction was performed with RIPA buffer containing protease and phosphatase inhibitors (2 mM Na_3_VO_4_ and 10 mM Na_4_P_2_O_7_), followed by protein quantification. Total cellular proteins (20 μg/well) were incubated with Laemmli sample buffer containing β-mercaptoethanol and boiled for 5 min, and subjected to 10% SDS/PAGE. Proteins were transferred to polyvinylidene fluoride (PVDF) membranes using a semi-dry system at 24 V for 1 h and 30 min. After transfer, the membranes were blocked with a 5% non-fat milk in TBS-T buffer (10 mM Tris-base, 150 mM NaCl, and 0.1% Tween 20) for 2 h at room temperature. After blocking, the membranes were incubated with primary Phospho-SAPK/JNK (Thr183/Tyr185) rabbit polyclonal antibody (9251) and Total-SAPK/JNK rabbit polyclonal antibody (9252) provided by Cell Signaling Technology (Beverly, MA, USA) for 16 h at 4 °C. Monoclonal anti-β-actin antibody (A5441) from Sigma-Aldrich (St. Louis, MO, USA) was used as a loading control. Horseradish peroxidase-conjugated goat anti-rabbit IgG secondary antibody from Cell Signaling Technology (Beverly, MA, USA) was used here. The immunoreactive bands were detected using Pierce ECL from Thermo Fisher Scientific (cat. number 32106). ImageJ software version 1.46r (National Institutes of Health, Bethesda, MD, USA) was utilized for protein expression analysis. β-actin and the total form of JNK were used for normalization.

### 4.10. Statistical Analysis

Thermal index analysis was used to evaluate the amplitude of thermal responses, calculated from the area under the curve (AUC). We established 36.5 °C as the baseline temperature and calculated the AUC from 2 to 6 h post-LPS. AUC values are expressed as degrees Celsius (*y*-axis) × hours (*x*-axis). Thermal index values and the other results (expressed as mean ± SEM) were analyzed using two-way ANOVA followed by Tukey’s multiple comparison test. GraphPad Prism version 10.5 (GraphPad Software, Inc., La Jolla, CA, USA) was used for statistical analyses. Values of *p* < 0.05 were considered statistically significant.

## 5. Conclusions

This study demonstrates that liraglutide exerts antipyretic effects through sexually specific mechanisms during systemic inflammation. In male rats, liraglutide reduces fever mainly by suppressing hypothalamic PGE_2_, the classical pyrogenic mediator. Conversely, in female rats, antipyretic effects occur independently of PGE_2_ modulation and instead correlate with reduced circulating IL-6, elevated hypothalamic 5-HT, and decreased JNK phosphorylation. These sex-specific responses reveal previously unrecognized sexual differences in GLP-1 receptor-mediated thermoregulatory and neuroimmune pathways. However, the correlational nature of these findings necessitates future mechanistic studies to establish causal relationships between observed biochemical alterations and temperature regulation. Given the widespread use of GLP-1 analogs for metabolic disorders, these findings suggest potential sex-dependent therapeutic effects during inflammatory conditions and highlight the importance of considering biological sex in evaluating GLP-1 analog responses. Future investigations should explore whether these sex-specific effects are also applicable to human physiology.

## Data Availability

Data is available in an online repository. The datasets generated and analyzed during the current study are available in the Zenodo repository at https://doi.org/10.5281/zenodo.17257376 (currently under review, will be publicly accessible upon publication).

## Abbreviations

The following abbreviations are used in this manuscript:

5-HT	Serotonin
AMPK	AMP-activated protein kinase
AUC	Area Under the Curve
CGRP	Calcitonin Gene-Related Peptide
CM^•^	3-methoxycarbonyl-2,2,5,5-tetramethylpyrrolidine-1-oxyl
CMH	1-hydroxy-3-methoxycarbonyl-2,2,5,5-tetramethylpyrrolidine
CP^•^	3-carboxy-proxyl nitroxide
COX-2	Cyclooxygenase-2
DA	Dopamine
ELISA	Enzyme-Linked Immunosorbent Assay
EP3	Prostaglandin E_2_ receptor 3
ERK	Extracellular signal-regulated kinase
ESA	Electrochemical detector brand (Coulochem III)
GLP-1	Glucagon-Like Peptide-1
GLP-1R	Glucagon-Like Peptide-1 receptor
HbNO	Nitrosylated Hemoglobin
HPLC	High-Performance Liquid Chromatography
iNOS	Inducible Nitric Oxide Synthase
i.p.	Intraperitoneal
JNK	c-Jun N-terminal kinase
LIRA	Liraglutide
LPS	Lipopolysaccharide
NF-κB	Nuclear Factor kappa B
NO	Nitric Oxide
PGE_2_	Prostaglandin E_2_
PVDF	Polyvinylidene Fluoride
RIPA	Radioimmunoprecipitation Assay buffer
RPMI	Roswell Park Memorial Institute medium
ROS	Reactive Oxygen Species
SAL	Saline
SDS/PAGE	Sodium Dodecyl Sulfate Polyacrylamide Gel Electrophoresis
SEM	Standard Error of the Mean
TBS-T	Tris-Buffered Saline with Tween 20
TGFβ1	Transforming Growth Factor beta 1
